# Understanding the Solid-State Structure of Riboflavin through a Multitechnique Approach

**DOI:** 10.1021/acs.cgd.4c00480

**Published:** 2024-07-18

**Authors:** Christopher J. H. Smalley, Colan E. Hughes, Mariana Hildebrand, Ruth Aizen, Melanie Bauer, Akihito Yamano, Davide Levy, Simcha K. Mirsky, Natan T. Shaked, Mark T. Young, Ute Kolb, Ehud Gazit, Leeor Kronik, Kenneth D. M. Harris

**Affiliations:** † School of Chemistry, Cardiff University, Cardiff, Wales CF10 3AT, U.K.; ‡ Department of Molecular Chemistry and Materials Science, Weizmann Institute of Science, Rehovoth, 76100, Israel; § The Shmunis School of Biomedicine and Cancer Research, George S. Wise Faculty of Life Sciences, Tel Aviv University, Tel Aviv 6997801, Israel; ∥ Center for High Resolution Electron Microscopy (EMC-M), Johannes Gutenberg University Mainz, Duesbergweg 10-14, Mainz 55128, Germany; ⊥ Rigaku Corporation, 3-9-12 Matsubara-cho, Akishima, Tokyo 196-8666, Japan; # Wolfson Applied Materials Research Center, Tel Aviv University, Tel Aviv 6997801, Israel; ∇ Department of Biomedical Engineering, Faculty of Engineering, Tel Aviv University, Tel Aviv 6997801, Israel; ○ School of Biosciences, Cardiff University, Cardiff, Wales CF10 3AX, U.K.

## Abstract

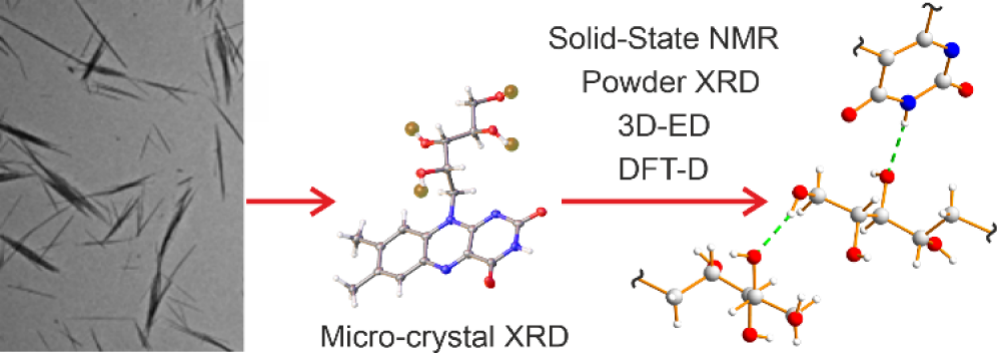

Crystalline riboflavin (vitamin B_2_) performs an important biological role as an optically functional material in the *tapetum lucidum* of certain animals, notably lemurs and cats. The *tapetum lucidum* is a reflecting layer behind the retina, which serves to enhance photon capture and vision in low-light settings. Motivated by the aim of rationalizing its biological role, and given that the structure of biogenic solid-state riboflavin remains unknown, we have used a range of experimental and computational techniques to determine the solid-state structure of synthetic riboflavin. Our multitechnique approach included microcrystal XRD, powder XRD, three-dimensional electron diffraction (3D-ED), high-resolution solid-state ^13^C NMR spectroscopy, and dispersion-augmented density functional theory (DFT-D) calculations. Although an independent report of the crystal structure of riboflavin was published recently, our structural investigations reported herein provide a different interpretation of the intermolecular hydrogen-bonding arrangement in this material, supported by all the experimental and computational approaches utilized in our study. We also discuss, more generally, potential pitfalls that may arise in applying DFT-D geometry optimization as a bridging step between structure solution and Rietveld refinement in the structure determination of hydrogen-bonded materials from powder XRD data. Finally, we report experimental and computational values for the refractive index of riboflavin, with implications for its optical function.

## Abstract 2

A multitechnique approach (based on microcrystal XRD, powder XRD, 3D-ED, solid-state ^13^C NMR, and DFT-D calculations) to investigate the solid-state structural properties of riboflavin reveals a new interpretation of the intermolecular hydrogen-bonding arrangement in this material. Experimental and computational values of the refractive index of this material are also reported, with implications for its optical function.

## 1 Introduction

Molecular crystals based on small organic molecules serve as functional materials in many optical systems, across a variety of organisms (see refs^1−3^ for recent overviews). Guanine assemblies in particular have been widely investigated and identified in many phyla.^1^ Optically functional crystals of guanine facilitate the production of structural colors in various organisms. Crystalline guanine is also utilized to construct mirrors in animal eyes, for image formation and enhancement of photon capture. Recently, reflective structures in the eyes of decapod crustaceans were found to be made of isoxanthopterin crystals.^4^ This molecule belongs to the pteridine family, members of which were previously known only as pigments.

Riboflavin (7,8-dimethyl-10-ribityl-isoalloxazine), also widely known as vitamin B_2_, is a mildly soluble essential vitamin that plays a crucial role in a wide range of metabolic pathways as a precursor to the essential cofactors flavin mononucleotide (FMN) and flavin adenine dinucleotide (FAD), which are utilized in numerous enzymatic reactions involving electron transfer.^5^ It is perhaps not as widely known that riboflavin, in crystalline form, has another important physiological role as an optically functional material identified in the *tapetum lucidum* of lemurs and cats.^6,7^ The *tapetum lucidum* is a reflecting layer that is positioned behind the retina in the eyes of vertebrates. Its purpose is to provide the photoreceptor cells with a second opportunity to absorb light that was not captured during its first passage, thereby enhancing photon capture and vision ability in low-light settings.^8^ The riboflavin structures do not only reflect light to the retina but also absorb short wavelengths and emit in the visible range of the spectrum, which is highly biologically relevant for the light-sensitive rhodopsin receptor.^9^ This dual functionality can also enhance photon capture in the *tapetum lucidum*. In the lemur, plate-like crystals of riboflavin have been extracted from the *tapetum lucidum*,^6^ whereas in the cat, fluorescent molecules identified as riboflavin have been extracted in the form of rod-like crystals.^7,9,10^ In each case, the crystal structure of the biogenic material has not yet been determined. Although studies of different solid forms of riboflavin (probably representing polymorphs and/or hydrate phases) were reported some time ago, these studies focused on optical measurements rather than characterization of structural properties.^11,12^

Both biogenic guanine^13^ and biogenic isoxanthopterin^4^ crystals are layered structures based on planar sheets of hydrogen-bonded molecules. Optical functionality is then primarily facilitated by the high refractive indices along specific crystallographic directions, which are generally attributed to the large polarizability of planar, conjugated molecules.^2^ Riboflavin, however, is structurally quite distinct from the planar guanine and isoxanthopterin molecules. In particular, the heterocyclic isoalloxazine ring in riboflavin is functionalized with a relatively long ribityl side chain, suggesting that riboflavin would not be able to form a planar hydrogen-bonded sheet structure of the type found in the biogenic structures of guanine and isoxanthopterin. Nevertheless, it has been observed recently that a new polymorph of isoxanthopterin (prepared synthetically) has high refractive indices in spite of the fact that the crystal structure is a nonplanar three-dimensional hydrogen-bonded network.^14^ Furthermore, it has been shown recently that crystals of 7,8-dihydroxanthopterin in the eyes of zander fish (*Sander lucioperca*) have a three-dimensional hydrogen-bonded network.^15^

The role of riboflavin as an optically functional material in biogenic systems and the absence of a published crystal structure of this material inspired us to attempt the crystallization of riboflavin, to determine the crystal structure, and then to further examine and analyze its optical properties. The procedures used to crystallize riboflavin in the present work yielded microcrystalline powder samples, which did not contain crystals of suitable size for structural characterization using conventional single-crystal X-ray diffraction (XRD). Instead, we have carried out an in-depth structural analysis based on a range of experimental and computational techniques, namely microcrystal XRD, powder XRD, three-dimensional electron diffraction (3D-ED), and high-resolution solid-state ^13^C nuclear magnetic resonance (NMR) spectroscopy, together with first-principles calculations based on dispersion-augmented density functional theory (DFT-D). While our investigation was in progress, an article was published independently by Guerain et al.,^16^ reporting a crystal structure determination of riboflavin from synchrotron powder XRD data (in conjunction with classical force-field calculations and periodic DFT-D calculations). The material prepared and studied in our work represents the same polymorphic form as that reported by Guerain et al., as the powder XRD pattern reported in their work matches the powder XRD pattern recorded for our material. However, while the crystal structure that we have determined (reported herein) is in broad agreement with that reported by Guerain et al., our structure differs in the intermolecular hydrogen-bonding arrangement, particularly concerning the hydrogen bonding involving the OH group at the end of the ribityl side chain. As discussed below, a range of experimental and computational techniques used in our investigation all support our structure as correctly describing the intermolecular hydrogen-bonding arrangement. Based on this revised crystal structure of riboflavin, we reflect on the role of riboflavin in optically functional crystals within a biological context, including experimental and computational studies of the refractive index of this material.

## 2 Results and Discussion

### 2.1 X-ray Diffraction and Electron Diffraction

**Figure 1:**
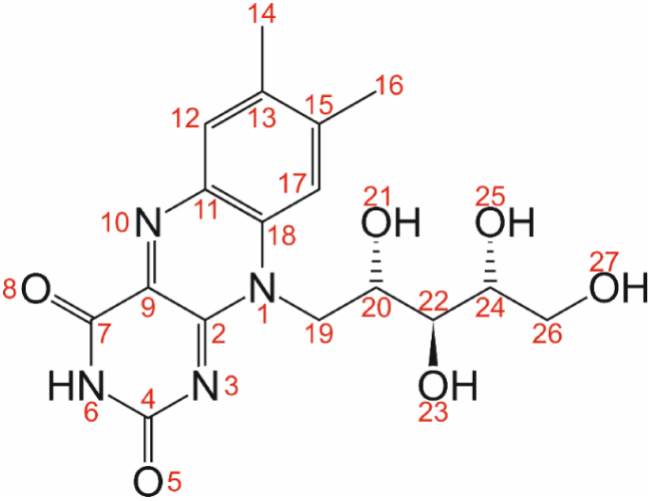
Riboflavin molecule with chirality {S, S, R} for the chiral centers C20, C22, and C24, respectively. The atom-numbering scheme shown is used throughout this article.

The molecular structure of riboflavin is shown in Figure 1, indicating the specific enantiomer found in biological systems and used in our studies, namely {S, S, R} for the chiral centers C20, C22, and C24, respectively. Throughout this paper, we refer to the atom-numbering scheme defined in Figure 1, both in our discussion of the structure reported in the present paper and in our discussion of the structure reported by Guerain et al.^16^

We first discuss results from our microcrystal XRD study of riboflavin, as this represents the most definitive approach for structure determination among the diffraction techniques used in our work. In our microcrystal XRD study, the crystal structure was determined at 293 K [*P*2_1_2_1_2_1_; *a* = 5.35270(10) Å, *b* = 15.1770(4) Å, and *c* = 20.1565(5) Å] and at 100 K [*P*2_1_2_1_2_1_; *a* = 5.3096(2) Å, *b* = 15.1057(5) Å, and *c* = 20.0284(7) Å], and these structures are deposited in the CSD. For all subsequent discussion and analysis in this paper, we consider these structures following transformation to the setting with *a* > *b* > *c*, as this setting facilitates comparison with the structure reported by Guerain et al.^16^ The transformation is defined by the following relationships between the unit cell vectors {***a***_o_, ***b***_o_, ***c***_o_} in our original setting and the unit cell vectors {***a***_t_, ***b***_t_, ***c***_t_} in the transformed setting: ***a***_t_ = −***c***_o_; ***b***_t_ = ***b***_o_; ***c***_t_ = ***a***_o_ (together with an appropriate origin shift). Cif files of the crystal structure of riboflavin determined from our microcrystal XRD data at 293 K and at 100 K in the transformed setting with *a* > *b* > *c* are available in Supporting Information (293 K, 100 K).

**Figure 2:**
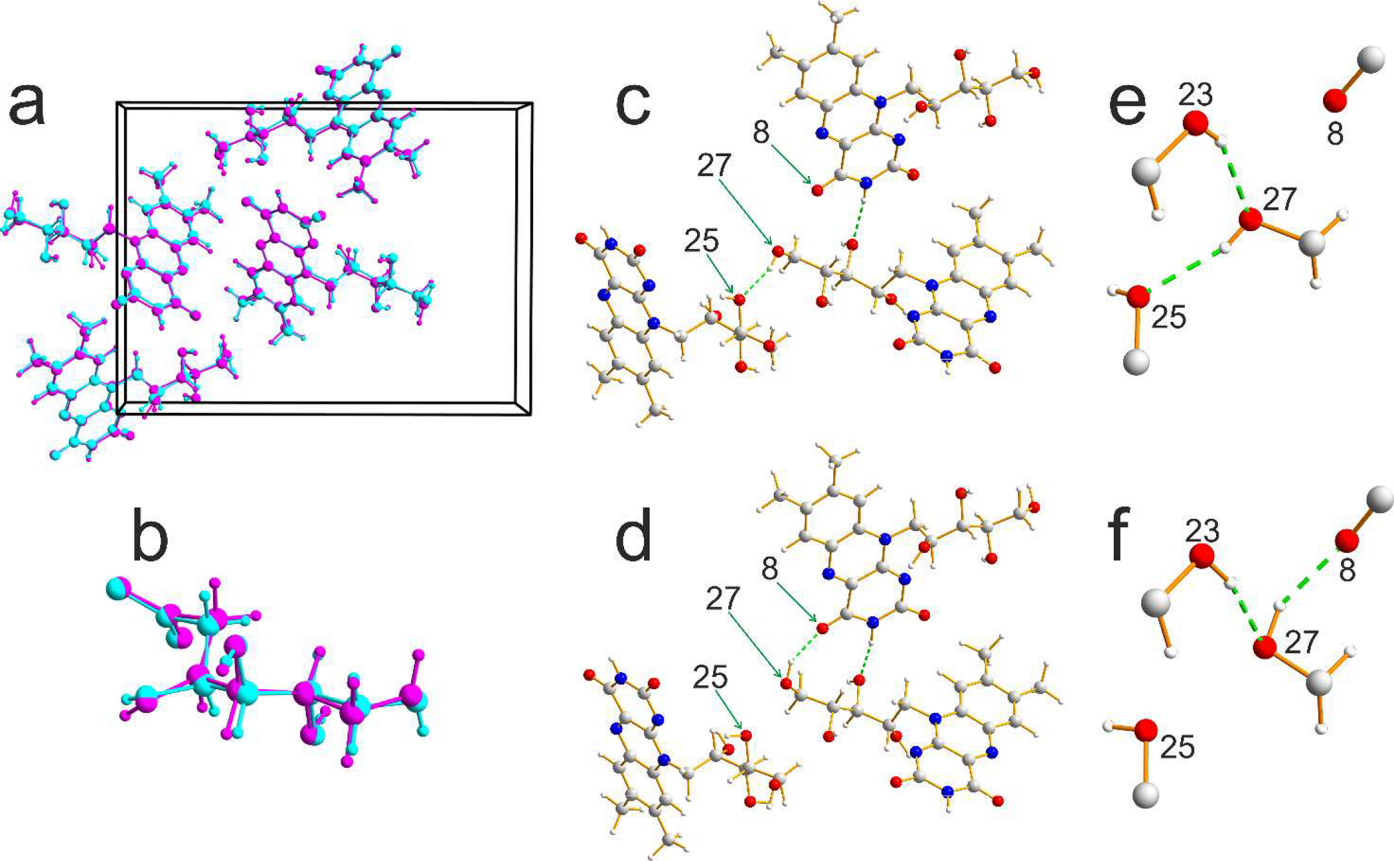
Comparison of the solid-state structures of riboflavin determined at 293 K in the present work from microcrystal XRD (structure A) and in ref^16^ from powder XRD (structure B). (a) Overlay of structure A (cyan) and structure B (magenta). (b) Overlay of the side chain of the riboflavin molecule (also including the C2–N1–C18 portion of the aromatic ring system) in structure A (cyan) and structure B (magenta), highlighting the conformational differences, particularly regarding the orientation of the terminal OH bond (right side of figure). (c, d) The intermolecular hydrogen-bonding arrangement between a given molecule (the central molecule shown) and two neighboring molecules in (c) structure A and (d) structure B, highlighting the different intermolecular hydrogen-bonding of the terminal CH_2_OH group (containing O27). (e, f) Expanded view of the hydrogen-bonding involving the terminal CH_2_OH group of the side chain of the riboflavin molecule in (e) structure A and (f) structure B. Hydrogen bonds are represented by green dashed lines.

First, we note that the crystal structures determined at 293 and 100 K in our microcrystal XRD study are identical (except for the effects of lattice contraction on cooling), indicating that no solid-state phase transition occurs between these temperatures. Our subsequent discussion is focused on the structure determined from microcrystal XRD analysis at 293 K (Figure 2), as this temperature matches those used in our powder XRD and solid-state NMR studies and in the powder XRD study reported by Guerain et al.^16^ Throughout the present paper, the structure determined in our work from microcrystal XRD at 293 K is denoted as structure A, and the structure reported in ref (16) is denoted as structure B.

Structure A and structure B have the same space group (*P*2_1_2_1_2_1_) and essentially the same unit cell (*a* ≈ 20.16 Å, *b* ≈ 15.18 Å, and *c* ≈ 5.35 Å). Furthermore, most of the atomic positions in the two structures are essentially identical (see Figure 2a,b), particularly concerning the aromatic ring system and the parts of the side chain closest to the aromatic ring. However, a significant difference between the two structures, highlighted in Figure 2c–f, concerns the hydrogen bonding of the terminal OH group of the side chain (containing O27). In structure A, the terminal OH group (O27) is the OH donor in an intermolecular O–H···O hydrogen bond with the OH group (O25) in the second-last position of the side chain of a neighboring molecule as the O acceptor. However, in structure B, the terminal OH group (O27) is the OH donor in an intermolecular O–H···O hydrogen bond with one of the C=O groups (O8) of the aromatic ring system of a different neighboring molecule as the O acceptor. The differences in the hydrogen-bonding arrangement are such that O27 is closer to the O25 neighbor than to the O8 neighbor in structure A, whereas O27 is closer to the O8 neighbor than to the O25 neighbor in structure B. As a consequence, small differences arise in the conformation at the end of the side chain in the two structures, as shown in Figure 2b. These structural differences are quantified in Table 1, which gives values of the torsion angles that define the conformation of the side chain and values of intermolecular O···O distances involving the terminal OH group (O27) of the side chain in the two structures. We note that, in both structure A and structure B, the terminal OH group (O27) is also involved as the O acceptor in an intermolecular O–H···O hydrogen bond with the OH group (O23) in the third-last position of the side chain of a neighboring molecule as the OH donor.

**Table 1:** Torsion Angles for the Ribityl Side Chain of the Riboflavin Molecule and the Intermolecular O···O Distances between the Terminal OH Group (Containing O27) and the O Atoms of Neighboring Molecules in Structure A and Structure B

	structure A (this work)	structure B (ref^16^)
Torsion Angle		
C2–N1–C19–C20	80.7°	89.4°
N1–C19–C20–C22	171.4°	171.1°
C19–C20–C22–C24	69.4°	60.0°
C20–C22–C24–C26	175.3°	175.8°
C22–C24–C26–O27	175.4°	161.7°
C24–C26–O27–H	79.7°	267.6°
O···O Distance		
O27···O25	2.97 Å	3.15 Å
O27···O8	3.11 Å	2.88 Å
O27···O23	2.94 Å	2.95 Å

While the hydrogen-bonding arrangement in structure A is assigned in the above discussion based on the intermolecular O···O distances, we now consider the capability to determine the positions of the H atoms of the OH groups involved in O–H···O hydrogen bonding directly from our microcrystal XRD data. In our analysis of the microcrystal XRD data, the positions of the H atoms of the OH groups were readily identified from peaks in the difference Fourier maps (see Figure S1), with peak magnitudes ranging from 0.31 e/Å^3^ to 0.36 e/Å^3^ at 293 K and from 0.38 e/Å^3^ to 0.42 e/Å^3^ at 100 K. In the final structure refinement calculations (from which the cif files deposited in the CSD were generated), the H atom of each OH group was refined using a riding model based on an idealized tetrahedral OH as a rotating group (AFIX 147 instruction in SHELXL). The final refined positions of these H atoms were close to the peaks observed in the difference Fourier map (see Figure S1); for example, for the H atom in the terminal OH group (containing O27), the distance between the final refined position and the corresponding peak in the difference Fourier map was 0.136 Å at 293 K and 0.069 Å at 100 K. Furthermore, structure refinement was also carried out involving unbounded refinement of the H-atom positions, with the refined H atoms of the OH groups remaining in positions close to those obtained using the riding model (AFIX 147), and with no significant changes to the intermolecular hydrogen-bonding arrangement. From this analysis, it is clear that the positions of the H atoms of the OH groups determined by structure refinement from our microcrystal XRD data, at both 293 and 100 K, are fully consistent with the assignment of the hydrogen-bonding arrangement characteristic of structure A discussed above based on consideration of O···O distances (Figure 2; Table 1).

**Figure 3:**
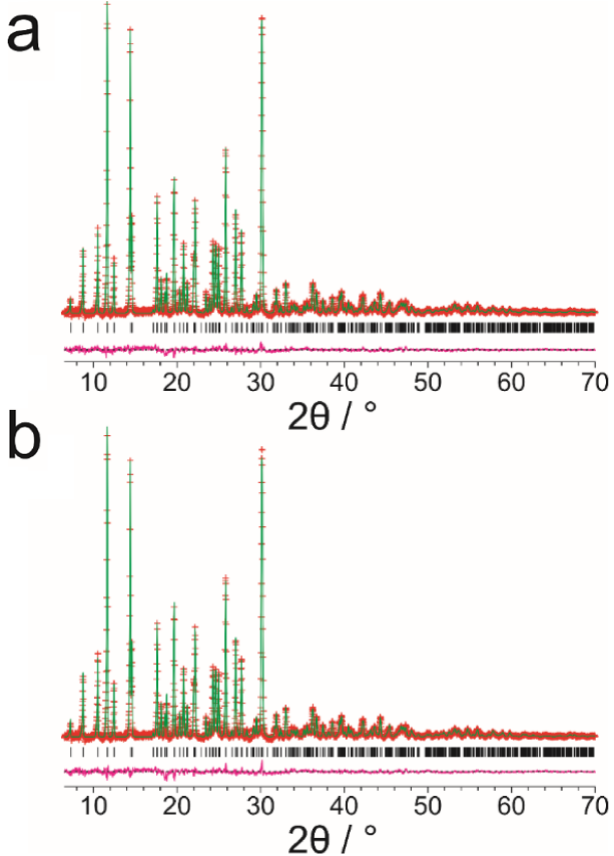
Final Rietveld refinements of the powder XRD data (background subtracted) for riboflavin using (a) structure A and (b) structure B as the structural model (red plus marks, experimental powder XRD data; green line, calculated powder XRD data; magenta line, difference between experimental and calculated powder XRD data; black tick marks, peak positions).

To assess the extent to which the differences between structures A and B are reflected in powder XRD data, we have carried out independent Rietveld refinement calculations using structure A and structure B as the starting structural model and in each case using the same powder XRD data set recorded at ambient temperature. The protocol for handling the Rietveld refinement calculations was kept identical in each case, and the hydrogen-bonding arrangement characteristic of structure A or structure B was preserved using appropriate restraints on the hydrogen-bond geometries. The final fits obtained in our Rietveld refinements for structure A and structure B are shown in Figure 3, and the final refined unit cell parameters for structure A [*a* = 20.0888(4) Å, *b* = 15.1271(4) Å, and *c* = 5.33472(10) Å] and structure B [*a* = 20.0886(4) Å, *b* = 15.1275(4) Å, and *c* = 5.33451(10) Å] are essentially the same as those reported by Guerain et al.^16^ for structure B [*a* = 20.01308(15) Å, *b* = 15.07337(12) Å, and *c* = 5.31565(4) Å]. Although our final Rietveld refinements for both structure A (Figure 3a) and structure B (Figure 3b) give a good quality of fit to the experimental powder XRD data, reflecting the fact that the two structures differ significantly only in a small region of the structure, the quality of fit is slightly better for structure A (*R*_p_ = 0.49%; *R*_wp_ = 0.66%) than for structure B (*R*_p_ = 0.52%; *R*_wp_ = 0.71%). On this basis, the Rietveld refinements from the powder XRD data support structure A as the more correct structural description.

**Figure 4:**
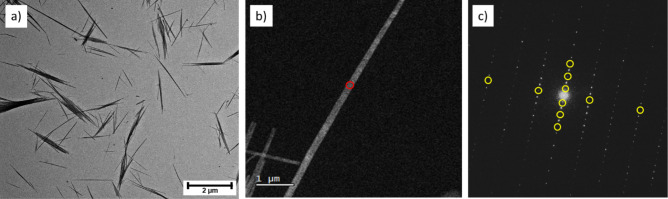
(a) Bright field TEM overview of the needle-like riboflavin crystals. (b) μ-STEM image of a single crystal selected for FAST-ADT acquisition (the red circle indicates the position of the electron beam in the diffraction experiment). (c) The (*h*0*l*) reciprocal lattice plane in the 3D-ED data with extinctions due to 2_1_ screw axes indicated by yellow circles. Projections of the 3D reconstruction are shown in Figure S3.

We have also determined the crystal structure of riboflavin from electron diffraction data recorded at 100 K on single microcrystals using the 3D-ED/FAST-ADT technique, with representative data shown in Figure 4. The collected data set comprised 5315 reflections (910 independent), corresponding to a completeness of 86% at a resolution of 0.7 Å. The unit cell (*a* = 20.25 Å, *b* = 15.25 Å, and *c* = 5.35 Å) and space group (*P*2_1_2_1_2_1_) determined from the 3D-ED data are fully consistent with the information established from our microcrystal XRD and powder XRD studies discussed above. Structure solution was carried out by direct methods using the program SIR2014^17^ and provided all non-H atoms and two H atoms (in methyl groups) at a final residual value of 25.7%. Kinematical refinement using SHELXL^18,19^ converged to *R* = 28%, *R*_w2_ = 61%, and GOF = 2.063. The H atoms in C–H bonds were added based on geometric considerations and refined using a riding model (AFIX); the H atoms in OH groups were found as residuals in the difference Fourier map and were fixed using the DFIX instruction in SHELXL (O–H distance, 0.96 Å).

Structure solution from the 3D-ED data was also carried out using the direct-space structure solution strategy implemented using a genetic algorithm (GA) in the program EAGER, which was originally developed for structure solution from powder XRD data^20−25^ and has recently been adapted for 3D-ED data.^26−28^ As shown in Figure S2, the structure solution giving the best fit to the 3D-ED data is in good agreement with structure A. In particular, recalling that O atoms are located much more reliably than H atoms in structure solution from 3D-ED data, we focus on analysis of the O···O distances in the region around the terminal OH group (O27) of the side chain. Specifically, the O···O distances between O27 and the three O atoms (O8, O23, and O25) in neighboring molecules that may potentially form O−H···O hydrogen bonds with O27 are: O27···O8, 3.25 Å; O27···O23, 2.94 Å; and O27···O25, 2.87 Å. These distances are much closer (see Table 1) to those characteristics of structure A than structure B, and thus, the structure solution obtained from the 3D-ED data provides further support to our assignment that the description of the hydrogen-bonding arrangement in structure A is correct.

### 2.2 DFT Calculations and Solid-State NMR Spectroscopy

In order to gain computational insights into the energetic properties of structure A and structure B, we have carried out geometry optimization of both structures using periodic dispersion-augmented DFT calculations (see Section 4 for details). Using Tkatchenko–Scheffler (TS) pairwise dispersion corrections,^29^ the optimized lattice parameters are *a* = 20.00 Å, *b* = 15.11 Å, and *c* = 5.34 Å for structure A (relative error compared to experimental values from microcrystal XRD at 100 K of 0.14%, 0.03%, and 0.57%, respectively) and *a* = 20.16 Å, *b* = 14.98 Å, and *c* = 5.43 Å for structure B (relative error compared to the same experimental values of 0.65%, 0.83%, and 2.27%, respectively). Clearly, the overall agreement between the experimental and DFT-D optimized lattice parameters is better for structure A, although the agreement is also reasonable for structure B. More importantly, however, the optimized structure A is more stable than the optimized structure B by ∼14.1 kcal/mol per unit cell (equivalent to ∼3.5 kcal/mol per molecule or ∼14.6 kJ/mol per molecule).

Because pairwise corrections may, in some cases, be insufficiently accurate for energy ordering of competing polymorphs,^30^ we have carried out additional optimization using many-body dispersion (MBD).^31,32^ From these calculations, the optimized lattice parameters were found to be *a* = 19.99 Å, *b* = 15.08 Å, and *c* = 5.33 Å for structure A (relative error compared to experimental values of 0.19%, 0.17%, and 0.38%, respectively) and *a* = 20.16 Å, *b* = 14.99 Å, and *c* = 5.43 Å for structure B (relative error compared to experimental values of 0.66%, 0.76%, and 2.27%, respectively). All trends seen with the TS calculations are conserved, and the optimized structure A is again in overall better agreement with experimental values. Significantly, the results from these calculations indicate that the optimized structure A is even more stable relative to the optimized structure B by ∼14.7 kcal/mol per unit cell (equivalent to ∼3.7 kcal/mol per molecule or ∼15.5 kJ/mol per molecule).

We note that, within the context of relative energies of experimentally observed polymorphs of organic materials, differences in energy are typically found to be lower than *ca*. 2.5 kcal/mol per molecule,^33^ suggesting that structure B may be unlikely to represent an experimentally accessible polymorphic form of riboflavin. Furthermore, based on the substantial difference in energy between structure A and structure B, no significant population of the hydrogen-bonding arrangement characteristic of structure B would be predicted to exist in the experimental system, for example, in a disordered crystal structure containing populations of both hydrogen-bonding arrangements. Indeed, inspection of difference Fourier maps in the analysis of our refinement of structure A from microcrystal XRD data provides no evidence for disorder in the hydrogen-bonding arrangement, such as the existence of a fractional population of the hydrogen-bonding arrangement characteristic of structure B.

**Figure 5:**
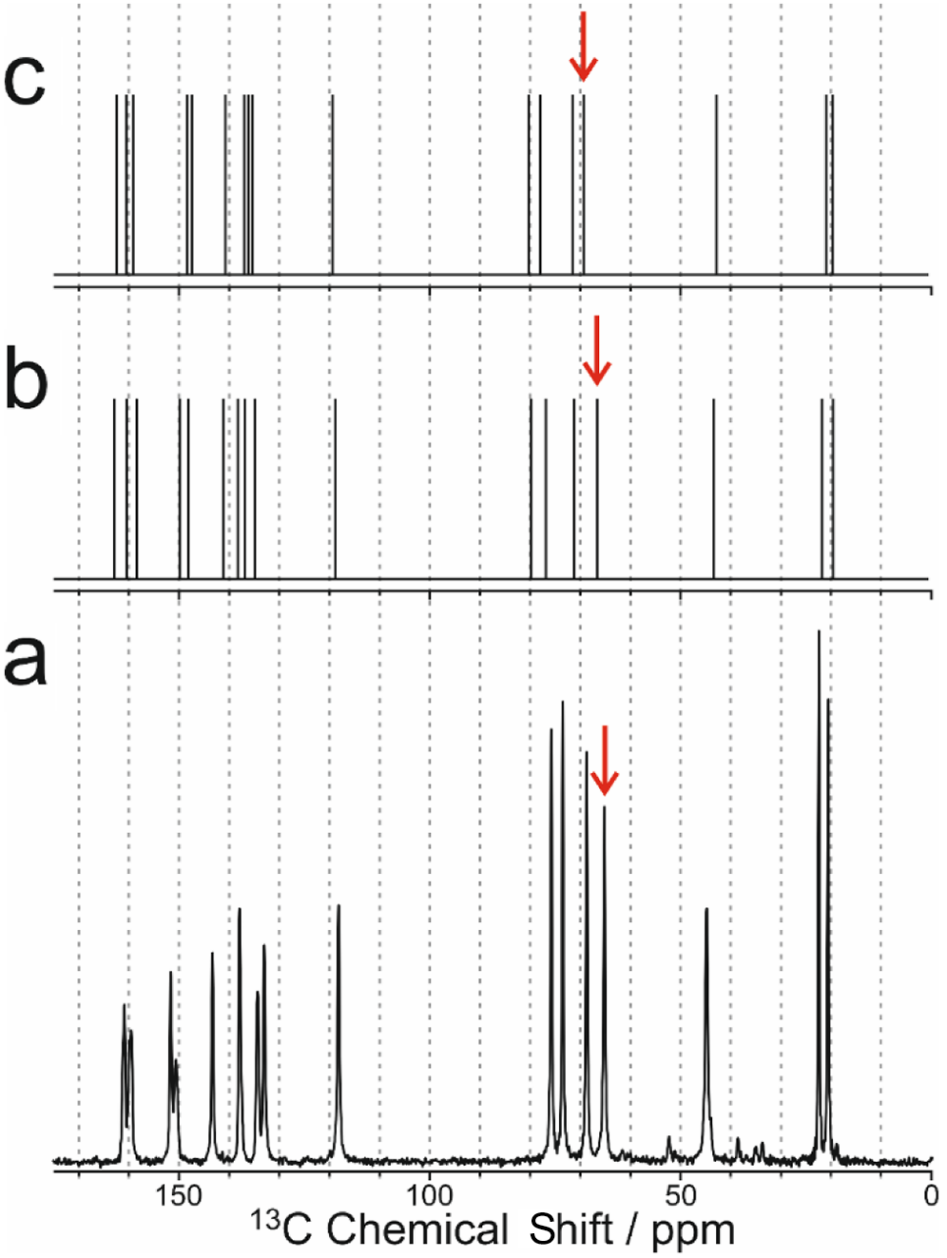
(a) The experimental high-resolution solid-state ^13^C NMR spectrum for riboflavin recorded at 293 K and (b, c) values of the isotropic ^13^C NMR chemical shifts calculated using DFT-GIPAW methodology for (b) structure A and (c) structure B. The red arrow in each spectrum indicates the isotropic peak for the ^13^C environment (C26) in the terminal CH_2_OH group of the side chain.

To obtain independent experimental evidence for the preferred hydrogen-bonding arrangement, we have recorded high-resolution solid-state ^13^C NMR data (Figure 5) for the same sample of riboflavin used in our microcrystal XRD and powder XRD studies. In addition, solid-state ^13^C NMR data have been calculated for structure A and structure B using DFT-GIPAW methodology^34−37^ in the program CASTEP.^38^ The calculated and experimental solid-state ^13^C NMR data are shown in Figure 5. In general, the calculated values of the isotropic ^13^C NMR chemical shifts are similar for structure A and structure B, but the overall match between the calculated and experimental values of the isotropic ^13^C NMR chemical shifts is better for structure A (RMSD = 1.96 ppm) than for structure B (RMSD = 2.54 ppm). However, as the main structural difference between structures A and B concerns the molecular conformation and intermolecular hydrogen-bonding arrangement involving the terminal CH_2_OH group (containing C26 and O27) of the side chain, we focus on the region of the solid-state ^13^C NMR spectrum corresponding to this CH_2_OH group, for which the experimental value of the isotropic ^13^C NMR chemical shift is δ_exp_ = 65.20 ppm. Significantly, there is substantially better agreement between the experimental (δ_exp_) and calculated (δ_calc_) values of the isotropic ^13^C NMR chemical shift for the terminal ^13^CH_2_OH group in the case of structure A (δ_calc_ = 66.14 ppm; δ_calc_ – δ_exp_ = 0.94 ppm) than structure B (δ_calc_ = 68.82 ppm; δ_calc_ – δ_exp_ = 3.62 ppm), lending further support to our conclusion that structure A represents the correct hydrogen-bonding arrangement.

### 2.3 Insights into the Determination of Hydrogen-Bonding Arrangements by Structure Solution from Powder XRD Data Combined with DFT-D Geometry Optimization

As all the experimental and computational evidence presented above supports structure A as the correct description of the solid-state structure of riboflavin, it is informative to consider how structure determination by analysis of powder XRD data coupled with DFT-D geometry optimization might lead to an incorrect description of the hydrogen-bonding arrangement corresponding to structure B. To assess this issue, we have carried out structure solution calculations using the same powder XRD data set used in our Rietveld refinement calculations discussed in Section 2.1. In this regard, we recall that structure solution is the stage of the process of structure determination from powder XRD data that comes after unit cell determination and before Rietveld refinement. Thus, the structure solution calculation starts with knowledge of the unit cell parameters (and space group) but with no knowledge of the arrangement of molecules within the unit cell, and the objective of structure solution is to generate a sufficiently good approximation to the correct structure to be suitable as a starting point for subsequent Rietveld refinement calculations.

**Figure 6:**
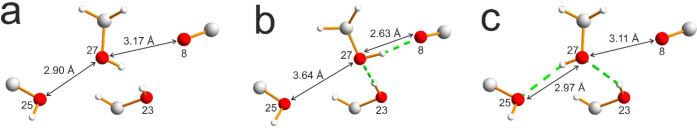
Local environment of the terminal OH group (containing O27) of the side chain of the riboflavin molecule in (a) the structure solution obtained directly from powder XRD data, (b) the structure obtained after applying DFT-D geometry optimization to the structure shown in (a), and (c) the correct hydrogen-bonding arrangement in structure A, determined from microcrystal XRD data. The O27···O25 and O27···O8 distances in each structure are indicated, and hydrogen bonds are represented by green dashed lines.

Our structure solution calculation was carried out using the direct-space genetic algorithm (GA) strategy implemented in the program EAGER^20−25^ (see Section 4 for details). As shown in Figure S4, the structure solution from our powder XRD data is very close to structure A but also relatively close to structure B, except for discrepancies in the region close to the end of the side chain. At the structure solution stage of the structure determination process, establishing the hydrogen-bonding arrangement based on the positions of H atoms is not reliable, given the intrinsic inaccuracy associated with locating H atoms in the analysis of powder XRD data. Thus, a more reliable approach is to assess the O···O distances. In our structure solution from powder XRD data, the distances between the O atom of the terminal OH group (O27) and the three O atoms (O8, O25, and O23) in neighboring molecules that may potentially form O–H···O hydrogen bonds with O27 are: O27···O8, 3.17 Å; O27···O23, 2.99 Å; and O27···O25, 2.90 Å. From these O···O distances (see Figure 6a), it is reasonable to deduce that O27 is involved in O–H···O hydrogen bonds with O25 and O23 in neighboring molecules, which matches structure A rather than structure B.

As DFT-D geometry optimization is commonly carried out at various stages of structure determination from powder XRD data as a basis for improving and validating the quality of the structural model, we carried out DFT-D geometry optimization (using the CASTEP program^38^ with fixed unit cell and using the PBE-TS method) on the structure solution obtained from our direct-space GA calculation and without any adjustment of the positions of the H atoms of the OH groups in order to construct a reasonable intermolecular hydrogen-bonding arrangement before carrying out the DFT-D geometry optimization. Following the DFT-D geometry optimization, we found that the terminal OH group (O27) had actually moved much closer to O8 than to O25 (O27···O8, 2.63 Å; O27···O25, 3.64 Å) and the O27–H bond was oriented toward O8 corresponding to the formation of an O27–H···O8 hydrogen bond (see Figure 6b). Thus, following DFT-D geometry optimization of the structure solution, the local structure around the terminal OH group actually corresponded to structure B.

We believe the reason that the DFT-D geometry optimization calculation nestled the structure into the hydrogen-bonding arrangement characteristic of structure B was because the terminal O27–H bond in the structure solution from powder XRD data was directed more toward the O8 neighbor than toward the O25 neighbor (see Figure 6a), even though the O27···O25 distance was significantly shorter than the O27···O8 distance, such that the DFT-D geometry optimization effectively pulled the O27–H group closer to O8 to form the O27–H···O8 hydrogen bond (see Figure 6b) corresponding to structure B, rather than rotating the O27–H bond around the C26–O27 bond to form the O27–H···O25 hydrogen bond characteristic of structure A (see Figure 6c).

The above analysis highlights a potential pitfall in the application of DFT-D geometry optimization as a bridging step after structure solution from powder XRD data and before Rietveld refinement. As H-atom positions in structure solution from powder XRD data are intrinsically unreliable, H atoms may be located in arbitrary positions that do not significantly affect the fit to the powder XRD data. However, the specific details of these positions can affect structural rearrangements (e.g., hydrogen-bond formation) that take place upon subsequent DFT-D geometry optimization. Therefore, when using DFT-D geometry optimization as a bridging step between structure solution and Rietveld refinement, it may be critical to adjust the positions of the H atoms to correspond to the most chemically and structurally reasonable hydrogen-bonding arrangement (based, for example, on considering the O···O distances in the structure solution) before carrying out the DFT-D geometry optimization. In the present case, when we adjusted the H-atom positions in this manner prior to DFT-D geometry optimization, the optimized structure corresponded to structure A.

While the discussion in this section highlights a scenario in which the use of periodic DFT-D geometry optimization calculations in conjunction with structure determination from powder XRD data may lead to an incorrect description of a hydrogen-bonding arrangement, we make no attempt to speculate upon the reasons why the procedure for structure determination of riboflavin reported by Guerain et al.^16^ (which was based on structure determination from powder XRD data together with DFT-D calculations) led to structure B rather than structure A.

### 2.4 Refractive Index of Riboflavin

Finally, recalling that riboflavin is used biologically as an optically functional material, we have calculated the refractive indices for structures A and B, yielding values of 1.65, 1.76, and 1.70 along the principal axes of structure A and 1.65, 1.69, and 1.77 along the principal axes of structure B. Thus, it is clear that the structural differences between structure A and structure B do not give rise to large changes in the optical properties. The computed results for structure A imply a scalar refractive index of ∼1.7, in excellent agreement with our experimentally determined values of 1.70 and 1.72 for two crystalline samples (see Methods and Figure S5). Importantly, while the refractive index of ∼1.7 for riboflavin is not as high as those of guanine (∼1.8)^13^ and isoxanthopterin (nearly 2.0),^14^ it is still sufficiently high to justify why cats and lemurs take advantage of a readily available biogenic molecule to perform a useful optical function. Moreover, biogenic guanine and biogenic isoxanthopterin are highly birefringent as a consequence of their layered structures based on planar hydrogen-bonded sheets, with a much lower out of plane refractive index (∼1.4). In contrast, the nonplanar three-dimensional hydrogen-bonded structure of synthetic riboflavin reported here gives rise to much more isotropic refractive behavior. Clearly, an important aim of future work is to determine the crystal structure and refractive indices of biogenic riboflavin and to establish their biological implications.

## 3 Concluding Remarks

Through the application of a multitechnique experimental and computational approach, we have established the correct description of the solid-state structural properties of riboflavin, particularly regarding the hydrogen-bonding arrangement, in comparison to the reported crystal structure published recently by Guerain et al.^16^ The crystal structure reported in ref^16^ (structure B) and the crystal structure reported in the present paper (structure A) represent the same polymorph of riboflavin and are in broad agreement with each other, but differ in the details of the intermolecular hydrogen bonding involving the OH group at the end of the side chain. In addition to evidence from various diffraction techniques (microcrystal XRD, powder XRD, and 3D-ED), the assignment of structure A as the correct description of the hydrogen-bonding arrangement is also supported by results from high-resolution solid-state ^13^C NMR spectroscopy and DFT-D calculations. Our DFT-D results indicate that structure A is more stable than structure B by *ca*. 3.7 kcal/mol per molecule. Based on this substantial difference in energy, no significant population of the hydrogen-bonding arrangement characteristic of structure B would be predicted to exist in the experimental system, in agreement with conclusions from our microcrystal XRD study, which show no evidence for disorder in the crystal structure.

As all our experimental and computational evidence supports structure A as the correct description, we have assessed in more general terms how it may be possible for structure solution from powder XRD data coupled with DFT-D geometry optimization (a strategy that is now commonly applied in structure determination from powder XRD data) to lead to an incorrect description of the hydrogen-bonding arrangement. Our analysis suggests that, while the positions of H atoms obtained in structure solution from powder XRD data are typically unreliable and often incorrect, the specific details of the H-atom positions can nevertheless have a critical influence on the structural rearrangements (e.g., hydrogen-bond formation) that may take place upon subsequent DFT-D geometry optimization. Thus, in structure determination of hydrogen-bonded materials from powder XRD data, when DFT-D geometry optimization is used as a bridging step between structure solution and Rietveld refinement, it may be critically important to adjust the positions of the H atoms to correspond to the most chemically and structurally reasonable hydrogen-bonding arrangement before carrying out the DFT-D geometry optimization.

Finally, we recall that the reported crystal structure of riboflavin was determined for a sample crystallized *in vitro* and does not necessarily correspond to the polymorphic form(s) of crystalline riboflavin present in biological systems. Clearly, an important target for future research is to focus on structure determination of the specific biogenic form(s) of crystalline riboflavin found in the *tapetum lucidum* of lemurs and cats. Indeed, given the contrasting morphological properties that have been reported for crystals of riboflavin extracted from the eyes of lemurs and cats, it is conceivable that they may actually represent different polymorphic forms.

## 4 Methods

### 4.1 Sample Preparation

The sample of riboflavin used in our microcrystal XRD, powder XRD, and solid-state NMR studies was (−)-riboflavin (≥98%) produced from *Eremothecium ashbyii* filamentous fungus and purchased from Sigma-Aldrich. An amount (0.0315 g) of this sample was added to preheated deionized water (50 mL; 80 °C) and allowed to dissolve for 20 min. The mixture was then hot-filtered to remove any excess solids. The solution was then placed in an incubator at 80 °C, and the temperature was decreased at a rate of 0.02 °C min^–1^. After reaching ambient temperature, the solution was transferred to a vacuum desiccator (containing dry silica as a desiccant) and left for several weeks. Each day during this period, the desiccant was dehydrated and the desiccator was evacuated. After 28 days, a sufficient amount of solid had precipitated as a microcrystalline powder sample, and the sample was collected and kept sealed under dry conditions to prevent hydration.

The sample of riboflavin for 3D-ED measurements was provided by BASF. The sample was suspended in distilled water at a ratio of 1:100 and then homogenized and deagglomerated using a vortexer. The sample was then diluted again 10-fold with distilled water, and an amount (3 μL) of the suspension was applied to a previously hydrophilized carbon-coated copper grid (300 mesh). A plasma cleaner was used to hydrophilize the TEM grid. The results from analysis of the 3D-ED data confirmed that this sample was the same polymorph of riboflavin as that used in the other studies reported here.

The sample of riboflavin for refractive index measurements was purchased from Sigma-Aldrich (purity >98%), and 1,1,1,3,3,3-hexafluoro-2-propanol (HFIP) 99% was purchased from Tzamal D-Chem. Riboflavin crystals were prepared using the solvent-switch method. A stock solution of riboflavin was prepared by dissolving riboflavin in HFIP to a final concentration of 12.5 mg/mL. Next, riboflavin was diluted in 99% methanol solution. Typically, 8–10 μL of riboflavin stock solution was dissolved in methanol to a final volume of 200 μL and left to crystallize overnight. Powder XRD confirmed that the material obtained by this crystallization method was in full agreement with the materials used in the other studies reported here.

### 4.2 DFT Calculations

DFT calculations were carried out using the Vienna Ab initio Simulation Package (VASP),^39^ based on the Perdew–Burke–Ernzerhof (PBE)^40^ functional and Tkatchenko–Scheffler (TS)^29^ pairwise dispersion corrections. Some additional calculations used the PBE functional with many-body dispersion (MBD).^31,32^ All structures were fully optimized, starting from experimentally determined coordinates and lattice parameters. An energy cutoff of 1000 eV was used along with a 3 × 3 × 3 *k*-point mesh. Gaussian smearing with a spread value of 0.05 eV was applied, and the system was relaxed to energy changes smaller than 10^–4^ eV. Refractive indices were determined by computing and then diagonalizing the dielectric tensor.

### 4.3 Microcrystal XRD

Microcrystal XRD data were recorded for crystals of riboflavin at 100 K (for a crystal with maximum and minimum dimensions of 100 and 15 μm, respectively) and at 293 K (for a crystal with maximum and minimum dimensions of 150 and 10 μm, respectively) on a Rigaku XtaLAB Synergy Custom FR-X diffractometer using a Rigaku HyPix-6000HE area detector and an Oxford Cryosystems cooling apparatus. This instrument was equipped with a microfocus rotating anode X-ray generator for CuKα radiation (λ = 1.54184 Å), with a multilayer confocal optics monochromator. Data collection and data processing were controlled using CrysAlisPro [Rigaku Oxford Diffraction, 2019]. Structure solution and structure refinement were carried out using SHELXT^41^ and SHELXL,^18^ respectively. Ideal geometry and riding coordinates were used for H atoms, with *U*_iso_ for the H atoms set to either 1.2 or 1.5 times the value for the atom to which they are bonded. Free rotation of methyl and OH groups were allowed during refinement. Specific details of the method for refinement of the H atoms of the OH groups are discussed in Section 2.1.

### 4.4 Powder XRD

The sample of riboflavin was ground and loaded into a glass capillary, which was then flame-sealed. High-quality powder XRD data suitable for structure determination were recorded at ambient temperature (21 °C) on a Bruker D8 diffractometer operating in transmission mode (Ge-monochromated CuKα_1_ radiation; Våntec detector covering 3° in 2θ; 2θ range, 4° to 70°; step size, 0.016°; data collection time, 86 h).

Le Bail fitting of the powder XRD data was carried out using the program GSAS^42^ (starting from the known unit cell parameters from our structure determined from microcrystal XRD data at 293 K), resulting in a good fit to the experimental powder XRD data (*R*_wp_ = 0.59%). The unit cell parameters and line shape parameters obtained in the Le Bail fitting process were used in subsequent Rietveld refinement. Independent Rietveld refinement calculations were carried out for a structural model corresponding to structure A and for a structural model corresponding to structure B. After initial refinement, both structures were subjected to DFT-D geometry optimization (using CASTEP^38^) with fixed unit cell parameters. Further Rietveld refinement was then carried out on the geometry optimized structures, with restraints applied to all hydrogen bonds based on the geometric properties of the DFT-D-optimized structures. No corrections for preferred orientation were required. In the final refinements, a good-quality fit to the powder XRD data was obtained for both structure A (Figure 3a) and structure B (Figure 3b), with a slightly higher quality of fit for structure A (*R*_p_ = 0.49%; *R*_wp_ = 0.66%) than for structure B (*R*_p_ = 0.52%; *R*_wp_ = 0.71%).

In connection with the investigations in Section 2.3, structure solution calculations using the same powder XRD data set were carried out using the direct-space strategy implemented using a genetic algorithm (GA) in the program EAGER.^20−25^ The contents of the asymmetric unit comprised one molecule of riboflavin, constructed using standard bond lengths and bond angles. Trial crystal structures were defined by a total of 15 structural variables, comprising three positional, three orientational, and nine torsion-angle variables. The torsion-angle variables were required to vary the conformation of the side chain, including variation of the positions of the H atoms of the OH groups by rotation around the C–OH bonds. A total of 40 independent GA structure solution calculations were carried out from a different random initial population in each case. Each GA calculation involved the evolution of a population of 100 trial structures for 100 generations, with 10 mating operations and 50 mutation operations carried out per generation. The quality of each trial structure was assessed from the figure-of-merit *R*_wp_ (which quantifies the level of agreement between the calculated powder XRD data for the trial structure and the experimental powder XRD data) and was used in the definition of the fitness function in the GA calculation. All 40 independent GA calculations generated essentially the same structure, and the trial structure giving the lowest value of *R*_wp_ was taken as the best structure solution.

### 4.5 3D-Electron Diffraction

The 3D-ED measurements were carried out on a FEI TECNAI F30 STWIN transmission electron microscope at an operating voltage of 300 kV equipped with a field emission gun. A condenser aperture (C2) of 10 μm, a gun lens 8, and a size 8 spot were selected to generate a semiparallel electron beam with a diameter of 200 nm for diffraction in nanobeam electron diffraction mode (NBED). Measurements were performed under cooling with liquid N_2_ in a Fischione tomography cryo sample holder in a tilt range of ±60°. STEM images of the crystals were taken with microprobe settings using an HAADF (High-Angular Annular Dark Field) detector from Fischione with the Digiscan unit from Gatan through Gatan Digital Micrograph software GMS3. Needle-like crystals with typical length of a few μm (see Figure 4a) were selected for diffraction experiments in NBED mode, with the data acquired using a CCD camera (16-bit 4096 × 4096 pixel Gatan ULTRASCAN4000). The use of μ-STEM for imaging and NBED mode for diffraction allowed a reduced electron dose to be applied to the sample, with diffraction data collected throughout the full-tilt range. In order to collect three-dimensional electron diffraction (3D-ED) data, a series of diffraction patterns were acquired by the automated electron diffraction tomography plugin (FAST-ADT) for Digital micrograph GMS3.^43^ To improve the integration quality of the diffraction intensities, the diffraction experiment was carried out with an electron beam precession (PED) movement controlled by a Digistar unit from NanoMEGAS SPRL.

For data processing in the eADT software,^44^ the diffraction data sets were converted to MRC format.^45^ Reconstruction of the 3D volume and determination of the unit cell parameters and space group were carried out using eADT. For extraction of intensities, both eADT and PETS2.0 were used.^46^ In PETS2.0, unit cell refinement is carried out automatically, while in eADT, the unit cell is adjusted manually. Structure solution by direct methods used the SIR2014 software,^17^ assuming the kinematic approximation *I* ≈ |*F*_hkl_|^2^. SIR2014 was also used to calculate difference Fourier maps. Scattering factors for electrons were taken from Doyle and Turner.^47^ Kinematical refinement of the crystal structure was carried out using SHELXL software.^18,19^

Structure solution from the 3D-ED data using the direct-space genetic algorithm (GA) strategy was carried out using the program EAGER.^26−28^ The procedure for these structure solution calculations was the same as that described above for structure solution from powder XRD data, with the exception that the quality of each trial structure was assessed using the figure-of-merit *R*_F_.

### 4.6 Solid-State ^13^C NMR Spectroscopy

High-resolution solid-state ^13^C NMR data were recorded at 9.4 T (^13^C Larmor frequency, 100.64 MHz) on a Bruker AVANCE III spectrometer using ramped ^1^H→^13^C cross-polarization (CP), magic-angle spinning (MAS; spinning frequency, 12 kHz), and ^1^H decoupling using SPINAL-64.^48^ The ^13^C NMR spectrum was referenced against the α polymorph of glycine,^49^ for which the carboxylate resonance was set to 176.5 ppm, corresponding to tetramethylsilane (TMS) as the primary reference.

Periodic dispersion-augmented DFT calculations to calculate the solid-state NMR chemical shifts for each crystal structure were carried out using the program CASTEP^38^ (academic release version 21.1.1), based on the PBE-TS approach, using a fixed unit cell, preserved space group symmetry, periodic boundary conditions, a basis set cutoff energy of 700 eV, and a uniform *k*-point grid of minimum sample spacing 0.05 × 2π Å^–1^. The isotropic ^13^C NMR chemical shielding values were calculated using a gauge-including projector augmented wave (GIPAW) approach,^34−37^ with a cutoff energy of 700 eV. From the isotropic ^13^C NMR shielding value (σ_calc_) calculated for each ^13^C environment in the crystal structure, the corresponding calculated isotropic ^13^C NMR chemical shift (δ_calc_) was determined from the equation:^36^
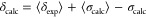
where ⟨δ_exp_⟩ denotes the mean of the isotropic ^13^C NMR chemical shifts determined from the experimental high-resolution solid-state ^13^C NMR spectrum and ⟨σ_calc_⟩ denotes the mean of the calculated isotropic ^13^C NMR shielding values. The value of ⟨δ_exp_⟩ was 107.078 ppm, with ⟨σ_calc_⟩ = 65.723 ppm for structure A and ⟨σ_calc_⟩ = 65.046 ppm for structure B.

### 4.7 Refractive Index Analysis

The scalar refractive index (RI) was determined for two crystalline samples of riboflavin using interferometric phase microscopy (IPM) with an illumination wavelength of 532 nm. Off-axis holograms of the samples immersed in a 99% methanol medium between two coverslips were acquired using a shearing IPM system^50^ and a supercontinuum laser source (NKT SuperK EXTREME) coupled to an acousto-optical filter (NKT SuperK SELECT). Background holograms were also acquired for the coverslips containing only methanol. Optical path delay (OPD) maps of the crystal samples were reconstructed from their respective holograms, with the background holograms used to remove field curvature and other phase aberrations. For illumination wavelength λ, the OPD at a given point (*x, y*) in the sample is defined by
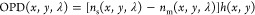
where *n*_s_ is the RI of the sample, *n*_m_ is the RI of the surrounding medium (i.e., the medium of the background hologram), and *h* is the sample height. The RI of the sample *n*_s_ can be determined from this equation as all other quantities are known. In this case, *n*_m_ is the known RI of methanol for the illumination wavelength λ.^51^ We note that *h* is known only along the central length of the crystal, where we assume that the local height is equal to the local diameter of the rod-like crystal, measured based on the OPD image.

## Data Availability

Additional supporting experimental data for this article may be accessed at http://doi.org/10.17035/d.2024.0325511442.

## Supporting Information Available

Additional figures showing results from difference Fourier analysis in the structure determination of the crystal structure of riboflavin from micro-crystal XRD data, the structure solution of riboflavin obtained from 3D-ED data, three-dimensional reconstruction of the reciprocal lattice from 3D-ED data, the structure solution of riboflavin obtained from powder XRD data, and OPD images for two crystals of riboflavin used in the determination of refractive index (PDF)
Cif file of the crystal structure of riboflavin determined from our micro-crystal XRD data at 293 K in space group P2_1_2_1_2_1_ transformed to the setting with *a* > *b* > *c*, as discussed in Section 2.1 (CIF)
Cif file of the crystal structure of riboflavin determined from our micro-crystal XRD data at 100 K in space group P2_1_2_1_2_1_ transformed to the setting with *a* > *b* > *c*, as discussed in Section 2.1 (CIF)

## Supplementary Material

cg4c00480_si_001.pdf

cg4c00480_si_002.cif

cg4c00480_si_003.cif

## Dedication

This paper is dedicated in honor of Professor Michael D. Ward (New York University) in recognition of his significant and wide-ranging contributions to materials chemistry and crystallization sciences.
