# Supplementary material for: Understanding the Solid-State Structure of Riboflavin through a Multitechnique Approach
Source: Cryst Growth Des. 2024 Jul 18;24(15):6256–66. doi: 10.1021/acs.cgd.4c00480 (PMC11311124; doi:10.1021/acs.cgd.4c00480)
Supplement: Supplementary file 1 — cg4c00480_si_001.pdf [file cg4c00480_si_001.pdf]

Supporting Information

Understanding the Solid-State Structure of Riboflavin through a Multi-technique Approach

Christopher J. H. Smalley,[1,+] Colan E. Hughes,[1] Mariana Hildebrand,[2] Ruth Aizen,[3,+] Melanie Bauer,[4] Akihito Yamano,[5] Davide Levy,[6] Simcha K. Mirsky,[7] Natan T. Shaked,[7] Mark T. Young,[8] Ute Kolb,[4,*] Ehud Gazit,[3,*] Leeor Kronik,[2,*] Kenneth D. M. Harris[1,*]

+ These authors contributed equally

* Authors for correspondence: HarrisKDM@cardiff.ac.uk; leeor.kronik@weizmann.ac.il; ehud.gazit@gmail.com; kolb@uni-mainz.de

1 School of Chemistry, Cardiff University, Cardiff CF10 3AT, Wales, U. K.
2 Department of Molecular Chemistry and Materials Science, Weizmann Institute of Science, Rehovoth 76100, Israel
3 The Shmunis School of Biomedicine and Cancer Research, George S. Wise Faculty of Life Sciences, Tel Aviv University, 6997801 Tel Aviv, Israel
4 Center for High Resolution Electron Microscopy (EMC-M), Johannes Gutenberg University Mainz, Duesbergweg 10-14, 55128 Mainz, Germany
5 Rigaku Corporation, 3-9-12 Matsubara-cho, Akishima, Tokyo 196-8666, Japan
6 Wolfson Applied Materials Research Center, Tel Aviv University, Tel Aviv, 6997801 Israel
7 Department of Biomedical Engineering, Faculty of Engineering, Tel Aviv University, Tel Aviv, 6997801, Israel
8 School of Biosciences, Cardiff University, Cardiff CF10 3AX, Wales, U. K.

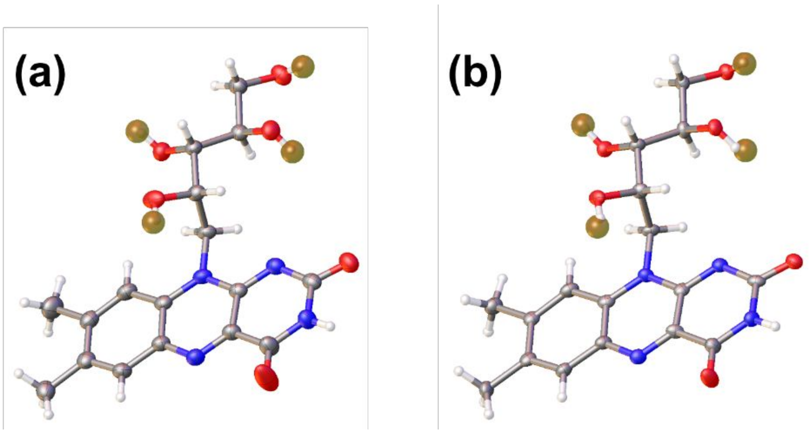

Figure S1. Results from difference Fourier analysis of the micro-crystal XRD data recorded for riboflavin at (a) 293 K and (b) 100 K. In each case, the molecule shown represents the asymmetric unit in the final refined crystal structure and the brown spheres indicate the location of the peak in the difference Fourier map representing the H atom of each OH group. The distance between the final refined position of each H atom (refined using a riding model with AFIX 147 in SHELX) and the corresponding peak in the difference Fourier map is: (a) at 293 K: O27H, 0.136 Å; O25H, 0.093 Å; O23H, 0.139 Å; O21H, 0.140 Å; and (b) at 100 K: O27H, 0.069 Å; O25H, 0.377 Å; O23H, 0.181 Å; O21H, 0.283 Å.

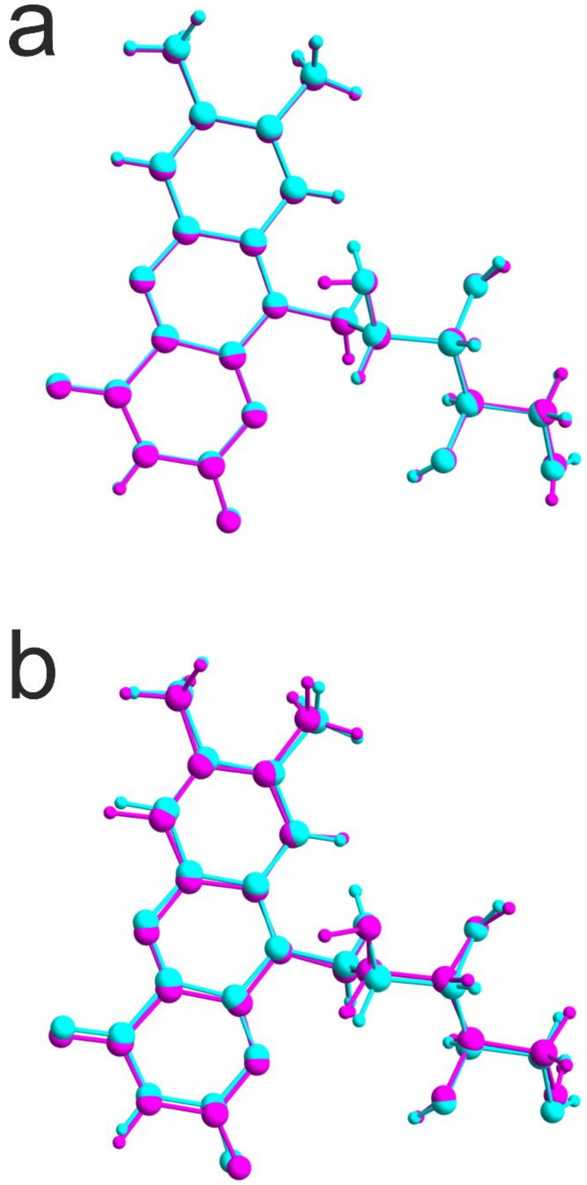

Figure S2. Overlays of the structure solution of structure A obtained from 3D-ED data (cyan) by direct-space structure solution using the program EAGER with: (a) the crystal structure (structure A) determined from our micro-crystal XRD study (magenta), and (b) the crystal structure (structure B) reported in ref. 16 (magenta). We note that the positions of the H atoms of the OH groups of the side-chain and the methyl groups attached to the aromatic ring are not structurally meaningful due to the intrinsic inaccuracy of locating H atoms in structure solution from 3D-ED data.

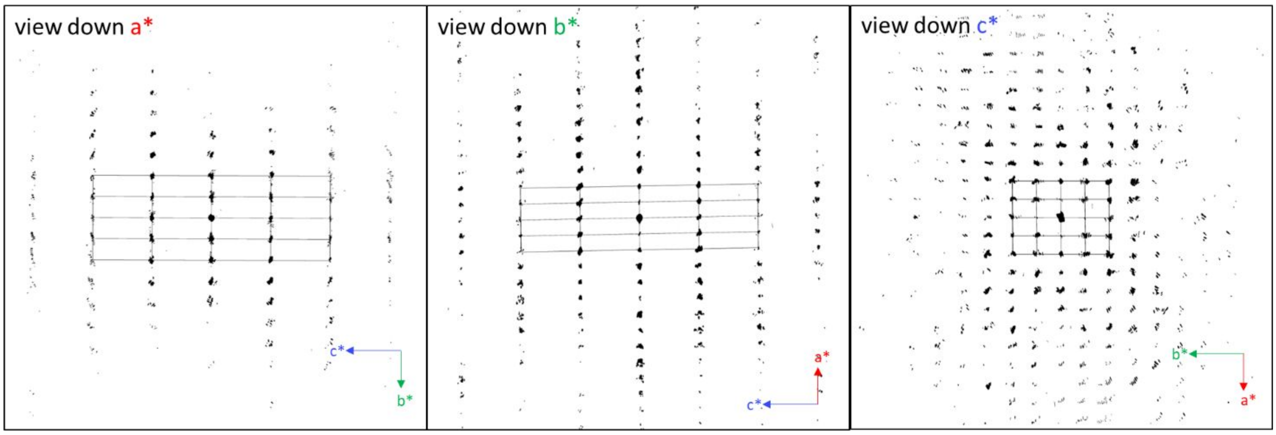

Figure S3. Three-dimensional reconstruction of the reciprocal lattice from the 3D-ED data recorded for riboflavin, viewed along *a**, *b** and *c**.

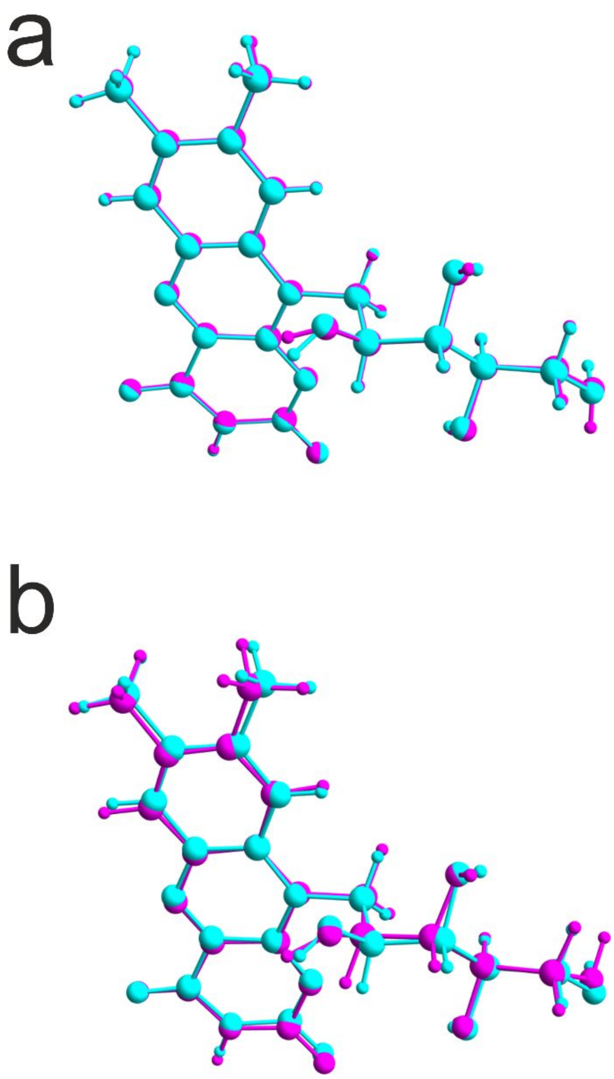

Figure S4. Overlays of the structure solution obtained from our powder XRD data (cyan) by direct-space structure solution using the program EAGER with: (a) the crystal structure (structure A) determined from our micro-crystal XRD study (magenta), and (b) the crystal structure (structure B) reported in ref. 16 (magenta). We note that the positions of the H atoms of the OH groups of the side-chain and the methyl groups attached to the aromatic ring are not structurally meaningful due to the intrinsic inaccuracy of locating H atoms in structure solution from powder XRD data.

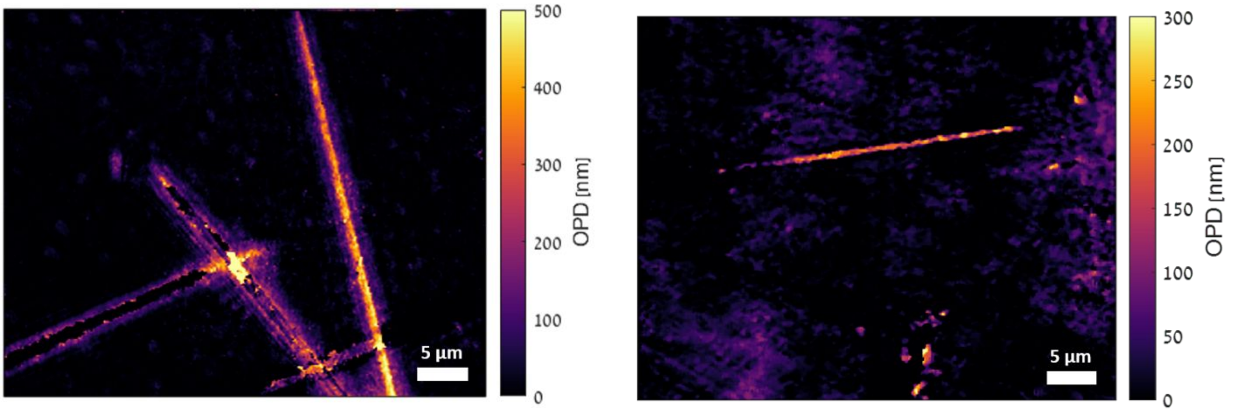

Figure S5. False colour OPD images for two crystals of riboflavin immersed in methanol. The method to determine the refractive index from these images is described in the Methods section of the main text. The two maps lead to scalar refractive index values of 1.72 (left) and 1.70 (right), with standard deviations of 0.11 and 0.05, respectively.
